# A novel *Enterococcus faecium* phage EF-M80: unveiling the effects of hydrogel-encapsulated phage on wound infection healing

**DOI:** 10.3389/fmicb.2024.1416971

**Published:** 2024-06-28

**Authors:** Mahshid Khazani Asforooshani, Ameneh Elikaei, Sahar Abed, Morvarid Shafiei, Seyed Mahmoud Barzi, Hamid Solgi, Farzad Badmasti, Aria Sohrabi

**Affiliations:** ^1^Department of Microbiology, Faculty of Biological Sciences, Alzahra University, Tehran, Iran; ^2^Department of Bacteriology, Pasteur Institute of Iran, Tehran, Iran; ^3^Department of Microbial Biotechnology, Faculty of Basic Sciences and Advanced Technologies in Biology, University of Science and Culture, Tehran, Iran; ^4^Isfahan Endocrine and Metabolism Research Center, Isfahan University of Medical Sciences, Isfahan, Iran; ^5^Department of Epidemiology and Biostatistics, Research Center for Emerging and Reemerging Infectious Diseases, Pasteur Institute of Iran, Tehran, Iran

**Keywords:** Bacteriophage, *Enterococcus faecium*, genome analysis, Hydrogel, wound infection

## Abstract

**Background:**

*Enterococcus faecium* is one of the members of ESKAPE pathogens. Due to its resistance to antimicrobial agents, treating this bacterium has become challenging. The development of innovative approaches to combat antibiotic resistance is necessary. Phage therapy has emerged as a promising method for curing antibiotic-resistant bacteria.

**Methods:**

In this study, *E. faecium* phages were isolated from wastewater. Phage properties were characterized through *in vitro* assays (*e.g.* morphological studies, and physicochemical properties). In addition, whole genome sequencing was performed. A hydrogel-based encapsulated phage was obtained and its structure characteristics were evaluated. Wound healing activity of the hydrogel-based phage was assessed in a wound mice model.

**Results:**

The purified phage showed remarkable properties including broad host range, tolerance to high temperature and pH and biofilm degradation feature as a stable and reliable therapeutic agent. Whole genome sequencing revealed that the genome of the EF-M80 phage had a length of 40,434 bp and harbored 65 open reading frames (ORFs) with a GC content of 34.9% (GenBank accession number is OR767211). Hydrogel-based encapsulated phage represented an optimized structure. Phage-loaded hydrogel-treated mice showed that the counting of neutrophils, fibroblasts, blood vessels, hair follicles and percentage of collagen growth were in favor of the wound healing process in the mice model.

**Conclusion:**

These findings collectively suggest the promising capability of this phage-based therapeutic strategy for the treatment of infections associated with the antibiotic-resistant *E. faecium*. In the near future, we hope to expect the presence of bacteriophages in the list of antibacterial compounds used in the clinical settings.

## 1 Introduction

*Enterococcus faecium* as a Gram-positive bacterium exhibits a remarkable tolerance to hostile conditions. It has a highly adaptable metabolism that allows it to grow in a variety of environments. *E. faecium* along with *Enterococcus faecalis* are common bacteria in the gastrointestinal tract (Pradal et al., 2023). However, *E. faecium* has acquired several capabilities that contribute to its success within hospital settings. These include an increasing resistance to antimicrobial agents that were previously considered first-line antibiotics (e.g., ampicillin, vancomycin and aminoglycosides) for the treatment of enterococcal infections, (Yim et al., 2017). In addition, *E. faecium* has acquired virulence genes that promote the formation of biofilm apparatus and improve its ability to colonize (Zhou et al., 2020). Biofilms are frequently encountered in the context of wound infections, and numerous studies have identified *Enterococcus* spp. as one of the most prevalent bacteria found in chronic wound infections. *Enterococcus* spp. has been consistently isolated from diabetic foot ulcers, venous leg ulcers, and burn wounds (Melo et al., 2019). The antibiotic susceptibility of entrapped bacteria in biofilms is significantly reduced due to metabolic and structural changes, leading to a 1000-fold reduction in antibiotic permeability. This situation exacerbates the eradication of infections associated with biofilm. High doses of antibiotics are leading to the development of antimicrobial resistance among bacteria, presenting a significant challenge to healthcare providers (Lebeaux et al., 2014; Vestby et al., 2020).

Recently, bacteriophages have become interesting alternatives for targeting superbugs and biofilm removal (Lin et al., 2017). Bacteriophages, also known as phages, are natural predators of bacteria that specifically replicate within bacteria. Notably, the potential of bacteriophages as therapeutic agents against bacterial infections, known as phage therapy, has sparked significant interest (Węgrzyn, 2022). Meanwhile, *Enterococcus* phages have attracted the attention of the biomedical research community as promising candidates for the development of therapeutics against drug-resistant enterococci (Canfield and Duerkop, 2020).

While the isolation of an lytic *Enterococcus* phage offers a promising initial step, translating this potential into a viable therapeutic requires *in vivo* evaluation in an animal model, where the complex interplay between the phage, host, and immune system can be accurately investigated (El Haddad et al., 2022). For proper release of the phage in wound site and to improve the healing process, design of a hydrogel based on sodium alginate (SA) and hyaluronic acid (HA) would be a promising route. Hydrogels are three-dimensional networks of hydrophilic polymers. They have substantial water absorption capability while retaining their structure. These compounds are being intensively investigated as wound dressings and drug depots for the sustained release of therapeutic agents (Yan et al., 2021). These properties make hydrogels interesting candidates for the delivery of bacteriophages in wound healing process, owing to their moisture retention, high adsorption capacity, biocompatibility, and ability to control the release of phages (Chang et al., 2021). In addition, the hydrophilicity of hydrogels minimizes cell adhesion and painless removal, offering advantages over conventional dressings (Shafigh Kheljan et al., 2023).

A study by Bean et al. observed a distinct zone of inhibition against *Staphylococcus aureus* using Phage K encapsulated within a hyaluronic acid methacrylate (HAMA)/agarose hydrogel system (Bean et al., 2014). Furthermore, Kaur et al. reported a significant reduction in bacterial biomass following treatment with a polyvinyl alcohol-sodium alginate (PVA-SA) hydrogel membrane encapsulated with a phage cocktail (MR10, Kpn5, and PA5). This treatment significantly reduced the amount of *S. aureus* bacteria by 6-log, *Klebsiella pneumoniae* by 6.37-log and *Pseudomonas aeruginosa* by 4.6-log after only 6 hours *in vitro* evaluation (Kaur et al., 2019). Treatment with an MR10-loaded hydrogel in a murine model of *S. aureus* burn injury led to both accelerated wound closure and a significant decrease in mortality rates. Additionally, Kumari et al. reported that the administration of phage Kpn5 encapsulated within an hydroxypropyl methylcellulose (HPMC) hydrogel significantly improved the percentage of survived animals which infected with *K. pneumoniae* compared to treatment with traditional antimicrobials like gentamicin (Kumari et al., 2011).

The aim of this study was the isolation and characterization of a lytic bacteriophage against clinical isolates of *E. faecium*. In addition, the morphological examination, determination of host range, ability to remove biofilms and other biological properties of the phage were evaluated. Finally, the therapeutic capability of the phage encapsulated in hydrogel on an *E. faecium* biofilm-induced wound infection model in mice was explored.

## 2 Methods and materials

### 2.1 Isolation of *Enterococcus faecium* strains

In this study, 50 clinical strains of *E. faecium* and the standard strain (*E. faecium* ATCC 1959) were considered. All clinical strains were isolated from patients in Imam Khomeini hospital in 2022 (Tehran, Iran) following standard protocols for bacterial culture and identification. Identification of *E. faecium* was confirmed by a combination of conventional biochemical tests and polymerase chain reaction (PCR) assay. The specific primers for *sodA* gene were previously reported as forward primer sequence (5′-GAAAAAACAATAGAAGAATTAT-3′) and the reverse primer sequence (5′-TGCTTTTTTGAATTCTTCTTTA-3′) (Alduhaidhawi et al., 2022). It should be noted that *E. faecium* strain used in all subsequent experiments was a clinical strain that originated from a lesion sample.

### 2.2 Antimicrobial susceptibility testing

The susceptibility of all isolates to various antibiotic classes was determined using two methods. The disk diffusion method, following the guidelines of the Clinical and Laboratory Standards Institute (Clinical and Laboratory Standards Institute [CLSI], 2023) was employed on Mueller-Hinton agar (Merck, Germany). A panel of ten antibiotics representing nine antibiotic classes was used: ampicillin-sulbactam (10/10 μg), streptomycin (300 μg), gentamicin (30 μg), chloramphenicol (30 μg), ciprofloxacin (5 μg), erythromycin (15 μg), linezolid (30 μg), rifampin (5 μg), teicoplanin (30 μg), and tetracycline (30 μg) (MAST Co., UK).

For vancomycin (Sigma-Aldrich, USA), the minimum inhibitory concentration (MIC) was determined using the broth microdilution method in sterile 96-well microtiter plates according to CLSI guidelines. Stock solutions of antibiotics were prepared in Mueller Hinton Broth and serially two-fold diluted from 256 to 0.5 μg/mL. Bacterial suspensions without antibiotics served as positive controls, while sterile medium represented negative controls. Each well was inoculated with a standardized inoculum of 1.5 × 10^8^ CFU/mL of the bacterial suspension. Following both disk diffusion and MIC procedures, plates were incubated at 37°C for 18–24 h. Interpretation of results for both methods followed based on established CLSI breakpoints.

### 2.3 Bacteriophage isolation and particle enrichment

Phage was collected and isolated from wastewater from Imam Khomeini hospital before chemical treatment. To remove bacterial cells and contaminants, the sample was centrifuged (8,000 rpm for 20 minutes) and filtered through a 0.22 μm membrane. To enrich the phage population, an equal volume of fresh Brain Heart Infusion (BHI) broth was added to the filtered effluent. This mixture was then inoculated with the fresh culture of *E. faecium* and incubated for 24 h at 37°C with shaking (150 rpm). After incubation, the new culture was centrifuged and filtered to separate the bacterial cells from the free phage particles. The double layer plaque assay (DLA), as described by Gong et al. (2016), was then used to concentrate and further purify the phage.

The enriched phage broth was serially diluted and the 0.1 ml aliquots from each dilution were co-incubated with 0.1 mL of *E. faecium* at 37°C for 10 min in a static incubator. This mixture was added to 5 ml of softened agar (0.4% agar) and then plated onto a solidified agar plate. After overnight incubation, clear zones (plaques) formed on the bacterial lawn, indicating areas where the phage lysed the bacteria. A single isolated plaque was picked and transferred to a sodium-magnesium buffer (100 mM NaCl, 8 mM MgSO4, 50 mM Tris-Cl) to be purified by two more rounds of plaque isolation.

### 2.4 Transmission electron microscopy

Transmission electron microscopy (TEM) was used to visualize the morphology of purified phage particles, following the method described by Lee et al. (2019). Briefly, 5 μl of the purified phage was fixed with 1% glutaraldehyde and placed on a Copper Mesh 400 grid coated with Formvar charcoal for a duration of 3–5 min. Subsequently, the sample was negatively stained with 2% PTA (phosphotungstic acid). The grid was then allowed to air-dry and subsequently analyzed using a Zeiss EM900 TEM apparatus (Carl Zeiss LEO EM 906 E, Germany) at a voltage of 50 kV.

### 2.5 Phage characteristics

#### 2.5.1 Host range determination

The host specificity of the isolated bacteriophage was assessed against 50 clinical *E. faecium* isolates. Bacterial susceptibility was evaluated using the spot test method. In brief, 10 μl of the purified phage suspension (7 × 10^14^ PFU/ml) was placed on a freshly cultured lawn of each bacterial strain and then incubated at 37°C for 24 hours in a static incubator. The procedure was replicated three times. The formation of plaques indicated susceptibility to the phage. In addition, the activity of the bacteriophage against other clinically relevant bacterial species, including *Enterococcus faecalis*, *Escherichia coli*, *Staphylococcus aureus*, *Streptococcus pneumoniae*, *Klebsiella pneumoniae* and *Pseudomonas aeruginosa*, was investigated to further determine the host range (Daubie et al., 2022).

#### 2.5.2 Environmental stability assay

To assess the sensitivity of phage to temperature ranges, 0.1 ml of selected phages with a titer of 7 × 10^16^ PFU/ml was inoculated into 0.9 ml of BHI broth. The suspensions were incubated for one hour at the following temperatures: −20°C, 4°C, 20°C, 37°C, 60°C, 70°C and 80°C in a static incubator. After incubation, the titer of the target phage was determined using the double-layer agar method (Litt and Jaroni, 2017).

The sensitivity of the phage to pH was investigated by preparing BHI broth media adjusted to the following pH values: 2, 4, 7, 10 and 14. Then 0.1 mL from the selected phage with titer of 7 × 10^16^ PFU/ml was added to 0.9 mL of the pH-adjusted culture medium in each medium. The suspensions were incubated at 37°C for one hour in a static incubator. The quantification of the phage titer was subsequently assessed through the utilization of the double-layer agar technique (Khawaja et al., 2016).

To investigate the stability of the phage particles in different NaCl concentrations, 0.1 mL of the phage (7 × 10^16^ PFU/ml) was added to 0.9 mL of BHI broth with different NaCl concentrations (5%, 10%, 15%). The suspensions were subjected to incubation at 37°C for an hour in a static incubator. The titer of the target phage was then determined using the double-layer agar method. Experiments were repeated three times for each condition.

#### 2.5.3 Determination of optimal multiplicity of infection (MOI)

Multiplicity of infection is the ratio of infecting or adsorbing phages to bacteria during infection (Abedon, 2016). For assessing the optimal MOI, the *E. faecium* strain was first grown in BHI broth at 37°C until early logarithmic growth phase (OD_625nm_ = 0.4). To achieve MOIs of 10, 1 and 0.1, different phage titers were prepared (10^9^ PFU/ml, 10^8^ PFU/ml, 10^7^ PFU/ml, respectively). Mixtures containing a bacterial growth of 1.5 × 10^8^ CFU/ml and various MOIs of phage were incubated with shaking (150 rpm) at 37°C for 4 h. The OD_625nm_ values were measured at 30-minute intervals and the experiment was done in triplicate. The MOI that resulted in the highest reduction in bacterial density was considered the optimal MOI (Ji et al., 2020).

#### 2.5.4 One-step growth culture and phage adsorption assay

To depict a one-step growth curve, EF-M80 phage (10^9^ PFU/ml) was mixed with host bacteria (1.5 × 10^8^ CFU/ml). This mixture was then incubated for 15 minutes at 37°C in a static incubator, followed by centrifugation at 10,000 rpm for 1 min. The supernatant was removed, and the remaining pellet containing phage-infected bacterial cells was re-suspended with 5 ml of fresh BHI broth. This suspension was then incubated with shaking at 150 rpm at 37°C. Aliquots were collected at 10-minute intervals, and their phage titers were immediately determined using the double-layer agar method. The experiment was repeated three times. The average burst size, quantified as the ratio of the phages formed during the rise period to the initial count of infected bacterial cells, was also determined (Wang et al., 2016). The rate of phage adsorption was detected using the double layer agar method. Host strain culture was exposed to bacteriophage at the titer of 7 × 10^16^ PFU/ml and incubated at 37°C. At predetermined time intervals (0, 5 and 10 min), 100 μl subsamples were collected from the suspension. These aliquots were then centrifuged to pellet bacterial cells. The resulting supernatants were assayed for unabsorbed bacteriophages at each time point. The procedure repeated for three times based on the protocol (Zhou W. et al., 2018) The phage adsorption rate determined as the ratio of the difference between the initial phage titer (P0) and the titer of unabsorbed phage at a given time point (Pt), to the initial phage titer (P0) (Pan et al., 2022).

### 2.6 Effect of bacteriophage on biofilm degradation

The biofilm plate assay, previously described, was utilized to assess the disruption of biofilm (Yazdi et al., 2018). In this assay, an overnight culture of *E. faecium* was diluted in BHI broth and transferred into 96-well flat-bottomed polystyrene tissue culture microtiter plates. Following incubation at 37°C for 24 h, the wells were discharged and washed with phosphate-buffered saline (PBS, pH = 7). Subsequently, the wells were treated with different dilutions of the phage particles from 10^1^ to 10^10^-fold and sterile physiological saline serving as the negative control. After 24 h of exposure at 37°C, the wells underwent washing with PBS followed by staining of the attached biofilm layer using Triphenyl Tetrazolium Chloride (TTC). The absorbance (at OD_480nm_) of each well was then measured. The lower OD showed more degradation of biofilm layers using phage intervention in a specific phage dilution. This method was applied for evaluation of the phage effect on 3-day and 5-day old of *E. faecium* biofilms and each experiment repeated for three times. Based on the data, the optimum phage dilutions were detected.

### 2.7 Phage genome sequencing and *de novo* assembly

DNA extraction was performed using a Viral Nucleic Acid Extraction Kit (FavorPrep™, Favorgen). Genome libraries were prepared and sequenced using the Illumina HiSeq 2000 platform (Illumina, Inc., San Diego, CA, USA). Paired-end sequence reads of 150 bp length were generated on an Illumina NextSeq (Micromon, Clayton, Australia) apparatus. The quality of the FASTQ paired-end files was checked using FastQC software (Brown et al., 2017). The trimming and filtering of the Fastq files was performed with the AfterQC (Chen et al., 2017). *De novo* assembly of short-read sequences was performed with QIAGEN CLC Genomics Workbench version 20. The quality of the assembly was assessed using the QUAST tool (Gurevich et al., 2013) and misassembles were corrected by the Mauve software (Darling et al., 2004). The *Enterococcus* phage genome sequence was annotated using the Prokka (rapid prokaryotic genome annotation) v1.14.5 (Seemann, 2014). Finally, the phage genome was submitted in the GenBank database via the BankIt submission route.

### 2.8 Comparative genome analysis

A dataset of related *Enterococcus* phages strain EF-M80, including 55 genomes (cut-off was coverage ≥ 70, and identity ≥ 70), was collected from GenBank and compared in the DNA and protein layers with the isolated *Enterococcus* phages. We conducted a distribution analysis of the accessory genomic elements using the ClustAGE (Ozer, 2018). The presence/absence matrix of DNA fragments is then converted into a dendrogram based on the unweighted pair-group method with arithmetic mean (UPGMA) using the Anvi’o tool (Eren et al., 2015). The dendrogram was visualized using the Interactive Tree Of Life (iTOL) platform and categorized based on the 50% threshold line (Letunic and Bork, 2021). In addition, the pan/core proteome analysis and the matrix of the present/absent genes were determined through the Roary (Rapid large-scale prokaryote pan-genome analysis) version 3.11.2 (Page et al., 2015). The inputs were annotated assemblies in GFF format generated by the Prokka version 1.14.5. The pan/core plot and dendrogram were depicted using the Python-based roary_plots.py tool.

### 2.9 Hydrogel preparation

Sodium alginate (SA) served as the primary natural polymer in this process. To fabricate the hydrogel, 4 g of SA was gradually dissolved in 100 ml of preheated (40°C) distilled water under continuous stirring to ensure a homogenous solution. The amount of 0.2 *g* of Carboxymethylcellulose (CMC) was dissolved by slow addition to 10 ml of distilled water with continues stirring to prevent aggregation. Hyaluronic acid (HA) was independently dissolved in deionized water at room temperature. Subsequently, all three solutions were combined, and the final mixture was autoclaved. Following sterilization and cooling, the solution was used to create hydrogel. The phage suspension was then thoroughly mixed with the hydrogel solution for 10 minutes to ensure homogenous phage distribution. To induce gelation, sterile 1% CaCl2 solution was subsequently incorporated into the mixture using a syringe. For film formation, the final solution containing phage and CaCl2 was immediately cast onto a plate and maintained at 20°C for 18-24 h. The solidified hydrogel film was then further cross-linked by adding 8 ml of 10% CaCl_2_ solution onto its surface. After one hour, excess liquid was discarded (Shafigh Kheljan et al., 2023).

### 2.10 Characterization of the hydrogel

#### 2.10.1 Determination of the swelling index

The hydrogel film was divided into 2 × 2 cm segments and allowed to dry at ambient temperature. Subsequent to determining the weight of the dried hydrogel (W_0_), it was exposed in distilled water and subsequently extracted at hourly intervals, as well as after a duration of 24 h. The excess water was eliminated by employing tissue paper, and the hydrogel was weighed (W_*S*_). The swelling index was determined using the designated formula (Gupta and Shivakumar, 2012)


Swellingindex(%)=[(W-SW)0/W]0×100

#### 2.10.2 Phage release

A hydrogel piece (2 × 2 cm) loaded with phage particles was aseptically transferred into a sterile culture tube containing BHI broth medium. The medium was supplemented with 1.5 × 10^8^ CFU/ml of *E. faecium* bacteria. The tube was then incubated at 37°C in a shaking incubator for 24 h. To determine phage release, the optical density (OD_625nm_) of the culture supernatant was measured. A control tube containing only BHI broth and bacteria, without the hydrogel, was processed identically for background subtraction.

#### 2.10.3 *In vitro* antibacterial activity assay

The antimicrobial activity of the hydrogels was assessed qualitatively via the disc diffusion method (Kaur et al., 2019). An overnight culture of bacteria, standardized to a 0.5 McFarland turbidity, was spread uniformly onto BHI agar plates using a sterile swab. Subsequently, disks were punched from both phage-loaded and phage-free hydrogels (negative control). These disks were then placed onto the solidified bacterial lawn and incubated at 37°C for 24 h. A clear inhibition zone surrounding each disk was evaluated after overnight incubation.

#### 2.10.4 Scanning electron microscopy (SEM) of hydrogels

Control and phage-loaded hydrogel films were characterized using scanning electron microscopy (SEM) to determine pore size and particle distribution and overall structure. Following complete dehydration, the hydrogels were sputter-coated with a thin layer of gold to enhance conductivity before SEM analysis (Martinez-Garcia et al., 2022).

#### 2.10.5 Fourier transform infrared spectroscopy (FTIR)

Fourier-transform infrared (FTIR) spectroscopy was employed to characterize the functional groups and bonding environment within the hydrogel samples. KBr pellet formation was utilized to prepare the samples for analysis. The infrared spectra were recorded in the range of 4,000–400 cm^–1^ using a spectrometer (Perkin–Elmer FTIR model 2000). The acquired spectra were then analyzed to identify characteristic absorption bands corresponding to the vibrational modes of functional groups present in the hydrogel (Mansur et al., 2008).

### 2.11 *In vivo* studies

*In vivo* experiments were conducted to examine the therapeutic efficacy of bacteriophage to evaluate the functionality of the designed hydrogel as a substitution for delivering the phage to the site of the wound infection. The study was authorized by the Ethics Committee of Al-Zahra University (Ir.ALZAHRA.REC.1401.015). Female BALB/c mice were obtained and given a week to acclimate to their new environment before commencing the experiments. To induce anesthesia, each mouse was injected intraperitoneal with 200 μl of ketamine/xylazine (in a 4:1 ratio). A bilateral full-thickness wound was created in the back skin of the mice using a 5 mm biopsy punch. The wound site was prepared by shaving the surrounding hair. Worth mentioning that the wound was created to a depth below the epidermis and superficial dermis, while avoiding damage to the muscles and minimizing bleeding with peak skin density. All wounds (except for the negative control group, which was treated with sterile distilled water) were infected with 50 μl of a 1.5 × 10^8^ CFU/ml suspension of *E. faecium* bacteria, and after 24 h, they were treated with the desired bacteriophage at optimal MOI of 0.1 (which in this case corresponds to 10^7^ PFU/ml) except for the positive control group. Following the experiments, each group of mice was placed in individual cages with sufficient availability to food and water. The mice were maintained in a 12-h light/dark cycle. All animals involved in this experiment received appropriate and compassionate care (Kaur et al., 2019). The wound healing process was monitored for over a period of 14 days. The mice were categorized into six groups, each consisting of five mice. All groups were as follows:

Healthy Control (HC): Unwounded, untreated mice.

Negative Control (NC): Wounded mice treated using sterile distilled water.

Positive Control (B): Wounded, *E. faecium*-infected, untreated mice.

Phage-Treated Group (Ph): Wounded, *E. faecium*-infected, phage-treated mice.

Phage Hydrogel Group (Ph-hyd): Wounded, *E. faecium*-infected, phage-loaded hydrogel-treated mice.

Hydrogel Control Group (Hyd): Wounded, *E. faecium*-infected, phage-free hydrogel-treated mice.

### 2.12 Histopathology studies

Following a 14-day observation period, the healed skin areas of the mice were excised and preserved in 10% formalin solution for subsequent pathological analysis. Hematoxylin and eosin (H&E) staining was performed to assess wound healing progression. The cell quantifying was done for neutrophils, fibroblasts, blood vessels, and hair follicles using the counting chamber. In addition, the percentage of collagen deposition and epidermal thickness were measured. To evaluate the wound-healing trajectory, standardized photographs were captured on days 3, 7, 10, and 14. Notably, this experiment was conducted concurrently with a separate comparative study that included healthy and uninfected wounded mice as the negative controls (Mansouri et al., 2022).

### 2.13 Statistical analysis

The data were tested for normal distribution using the Shapiro–Wilk test for small sample size. Groups were compared using a one-way analysis of variance (ANOVA) or multiple *t*-tests. *P*-values from statistical analyses were obtained, and *P* < 0.05 was considered statistically significant. GraphPad Prism software version 10 was employed for analyzing the data.

### 3 Results

#### 3.1 Bacterial strains

A total of 50 *E. faecium* strains were isolated from hospital patients and identified to the species level by biochemical tests and confirmed through PCR assay. The percentage of antibiotic resistance of the isolates was as follows: ampicillin-sulbactam (92%), streptomycin (100%), gentamicin (92%), vancomycin (100%), chloramphenicol (16%), ciprofloxacin (100%), erythromycin (100%), linezolid (6%), rifampin (100%), teicoplanin (70%), and tetracycline (72%). Antibiogram profile of bacterial isolates is available in the (Supplementary Table 1). The *E. faecium* strain serving as the host for EF-M80 phage isolation and propagation was designated EF.11 within our collection. Notably, EF.11 originated from a clinical lesion sample and displayed the highest level of sensitivity towards EF-M80, as evidenced by the formation of numerous clear plaques. Due to these observations, EF.11 was employed for subsequent experiments, including those investigating biofilm formation and *in vivo* efficacy.

#### 3.2 Phage isolation and morphology

**FIGURE 1:**
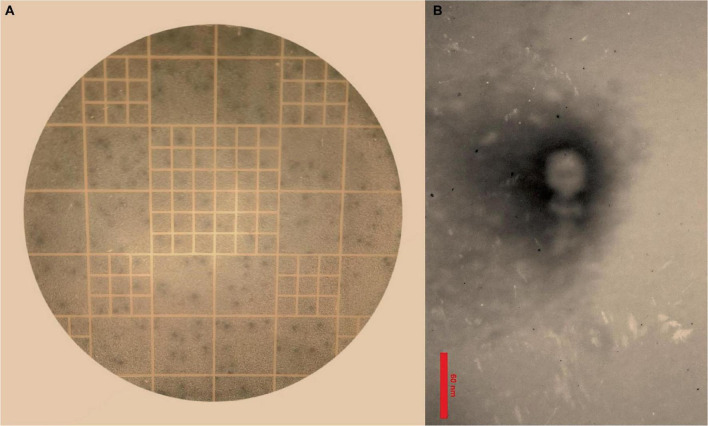
Isolation of *Enterococcus* phage strain EF-M80 from hospital sewage. **(A)** Plaque formation on double layer agar plate is showing the lytic activity of the phage against *E. faecium*. **(B)** Electron microscopy of the isolated phage negatively stained with 2% PTA at the scale bar of 60 nm. EF-M80 phage has icosahedral head and a collar structure consists of six long fibers.

The results of the DLA assay demonstrated that the purified phage, which has been named *Enterococcus* phage EF-M80, is effective against *E. faecium* (Figure 1A). Plaques were observed at a dilution of 10^14^-fold, indicating a high concentration of isolated phage in the initial stock. An estimated 7 × 10^16^ PFU/ml were present based on the average of 70 plaques observed at this dilution. The morphological characteristics of EF-M80 were represented through TEM, which revealed an icosahedral head and tail with approximate 35 nm in diameter and the length of 53 nm, respectively (Figure 1B). These findings, along with genomic analysis, suggest that EF-M80 belongs to the *Efquatrovirus* genus within the class of *Caudoviricetes*, as determined by the ICTV guidelines (Fauquet, 2005).

### 3.3 Phage characteristics

#### 3.3.1 Host range of the phage

Host range analysis was performed using a spot test to determine the phage’s lytic activity against a panel of thirty *E. faecium* clinical isolates. Sixty percent of the strains exhibited susceptibility, suggesting a broad host range within *E. faecium*. The phage demonstrated no lytic activity against other bacterial species such as *E. faecalis*, *E. coli*, *S. aureus*, *S. pneumoniae*, *K. pneumoniae* and *P. aeruginosa.*

#### 3.3.2 Phage stability tests

**FIGURE 2:**
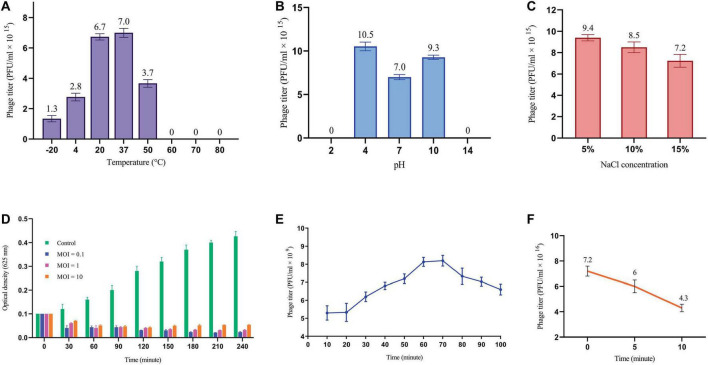
Phage replication cycle and its stability in different environmental conditions. **(A)** Phage titer changes at different temperatures. All differences in phage titers were statistically significant except group 20°C vs. 37°C. **(B)** The effects of different pH on phage titer. All differences were statistically significant except pH 2 vs. 14. **(C)** Lytic activity of the phage at high concentration of NaCl. All differences in phage titers were statistically significant except group with the concentration of 5% vs. 10%. See Supplementary Table 2. **(D)** MOI determination graph. **(E)** One-step growth cure revealing information about the life cycle of the phage. **(F)** The titer of non-adsorbed phage particles showing adsorption rate of almost 40% within 10 minutes.

The effects of environmental conditions on the stability of phage were investigated, including temperature, pH and salt concentration. The optimal temperature for phage stability was found to be 37°C, with the detectable stability between 4°C and 50°C (Figure 2A). Phage exhibited a broad pH tolerance and remained active from pH = 4 to 10 range (Figure 2B). However, stability decreased at both extremes of the pH range, reaching zero at pH = 2 and 14. The phage was demonstrated to remain stable over a range of NaCl concentrations of 5%, 10% and 15%, as illustrated in Figure 2C.

#### 3.3.3 MOI determination of the phage

A sufficient MOI is necessary to ensure efficient bacterial lysis, ultimately impacting the therapeutic efficacy of phages. As depicted in Figure 2D, all three MOIs showed a significant impact on the host cell population contrasted with the control group. Interestingly, the most reduction in bacterial growth was observed at MOI = 0.1, yet the differences among tested MOIs were not statistically significant. This finding suggests that multiple rounds of infection may be necessary to infect all of the bacterial cells in the culture.

#### 3.3.4 One-step growth and phage adsorption rate

The one-step growth curve analysis revealed a latent period of approximately 20 min and a total phage multiplication cycle of 60 min for this bacteriophage. In addition, the burst size was determined to be 12.6 PFU/infected cell (Figure 2E). Notably, the phage adsorption rate curve indicated that nearly 40% of the phages were adsorbed by the bacterial population within the initial ten minutes (Figure 2F). Understanding these key parameters is crucial for optimizing phage fitness, achieving a balance in early infection dynamics, and ultimately, for the successful implementation of phage therapy.

### 3.4 Biofilm degradation properties of the phage

Our findings demonstrate that phage treatment resulted in a decrease in absorbance within the wells compared to the control, indicating effective biofilm disruption. The phage dilution exhibiting the lowest absorbance, relative to the control, was designated as the most effective concentration for biofilm eradication. For one-day biofilms, the most effective phage dilution was 10^5^-fold. This value decreased to 10^4^-fold and 10^3^-fold for three-day and five-day biofilms, respectively (See Supplementary Table 3). These results suggest a positive correlation between biofilm maturity and the required phage concentration for an effective eradication.

### 3.5 Phage genome and proteome characterization

**FIGURE 3:**
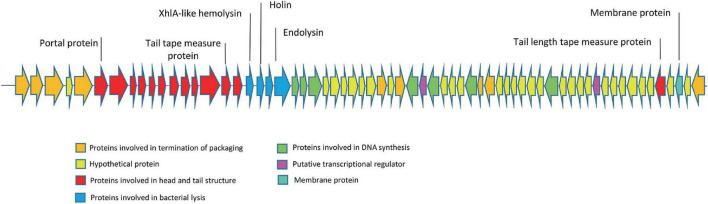
The genome structure of *Enterococcus* phage strain EF-M80. Hypothetical proteins and proteins involved in DNA replication were distributed across the genomes. The lytic cassette of EF-M80 (shown in blue) comprises enzymes (endolysins, holin) and a putative toxin (XhlA-like hemolysin) that act cooperatively to mediate phage-induced host cell lysis and release of progeny virions.

The genome of the EF-M80 phage has a total length of 40,434 bp and harbors 65 open reading frames (ORFs) with a GC content of 34.9% (GenBank accession number is OR767211). The genomic structure is illustrated in Figure 3, which displays their gene categorization into seven main groups: DNA packaging termination, head and tail structure, bacterial lysis, DNA replication, transcriptional regulation factors, membrane proteins, and hypothetical proteins. Six proteins were selected for analysis of sequence similarity among 56 *Enterococcus* phages. The portal protein and XhlA-like hemolysin were found to have 100% coverage and identity with other *Enterococcus* phages (see Supplementary Table 4).

**FIGURE 4:**
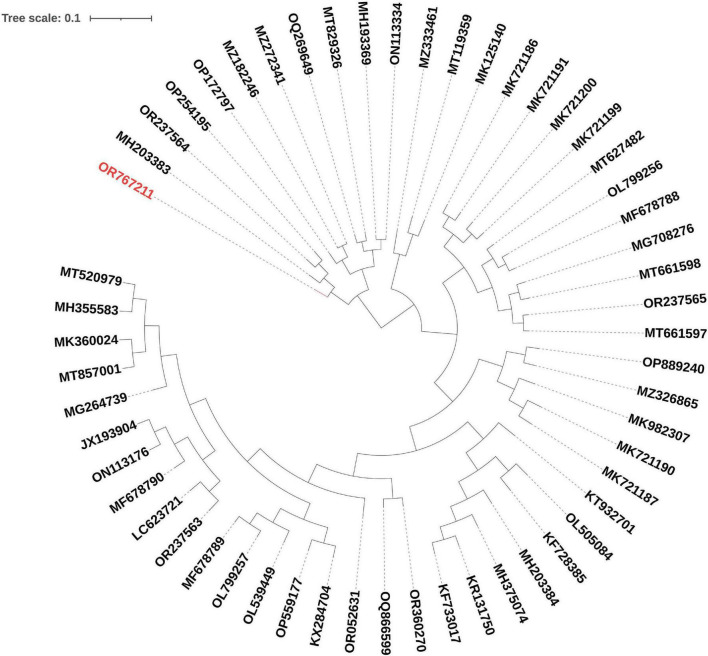
Phylogenetic positioning of EF-M80 phage within the *Enterococcus* phage landscape. A dendrogram constructed using the ClustAGE software depicts the evolutionary relationships between phage EF-M80 (colored in red) and other 55 *Enterococcus* phage genomes. The analysis revealed EF-M80’s closest relatives (MH203383) within the cluster, providing insights into its potential origins and evolutionary trajectory.

**FIGURE 5:**
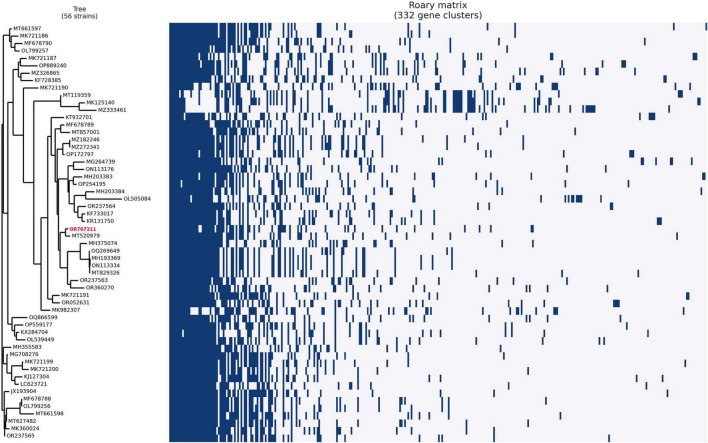
Unveiling the *Enterococcal* phage accessory genome: a pan/core proteomic analysis. This dendrogram shows the clustering patterns of 55 *Enterococcus* phage and EF-M80 phage (accession number in red color) proteomes generated using the Roary software. The analysis categorized the phage genomes into 332 gene clusters including core, accessory and unique components, revealing the common essential genes (core) and highlighting the diversity of accessory genes encoding auxiliary functions.

The phylogenetic analysis of EF-M80 with other *Enterococcus* phage genomes revealed that it was most closely related to phage vB_EfaS_AL3 with accession number MH203383, with 90% coverage and 97.61% identity (Figure 4). The clustering and distribution analysis of phages’ accessory genomic elements showed seven clades in DNA comparison, while a pan/core plot by Roary (sequence identity cut-off = 80%) revealed 332 gene clusters in proteome comparison. Pan/core analysis showed that a total of 10 proteins belong to the core region, while 97 proteins are in the shell (8 < genome < 53), and 225 proteins belong to the cloud (genome < 8). The neighbor-joining phylogenetic dendrogram of the genomes based on the presence/absence gene matrix revealed a high level of diversity among the *Enterococcus* phages (Figure 5).

### 3.6 Preparation and characterization of the hydrogel carrying EF-M80 phage

#### 3.6.1 Swelling ratio

**FIGURE 6:**
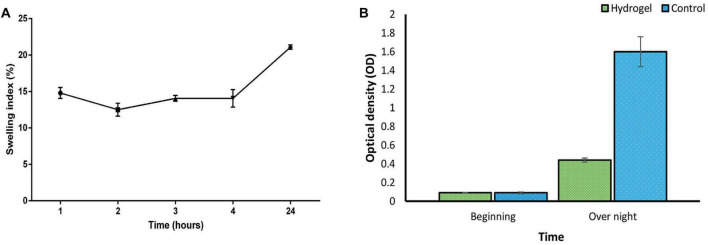
**(A)** Swelling index of SA/CMC/HA hydrogel showing a gradual increase in hydrogel mass with the most water uptake of 20% after 24 h. Swelling of the hydrogel triggers an expansion of the polymer network. **(B)** Suppression of bacterial growth in liquid culture by the phage-loaded hydrogel (green) compared to the control group without the hydrogel (blue). These two groups were statistically significant in overnight situation (*P*-value < 0.0001). See Supplementary Tables 5, 6 for more details.

As depicted in Figure 6A, the hydrogel’s swelling behavior aligns with previous reports (Shafigh Kheljan et al., 2023). Following immersion for 24 hours, the hydrogel demonstrated a swelling index of 20%. This enhanced swelling capacity is expected to facilitate the sustained release of the phage. However, it is crucial to maintain a balance, as excessive swelling can compromise the hydrogel’s structural integrity. Therefore, hydrogels exhibiting moderate swelling characteristics may be most optimal for wound healing applications (Zhou Q. et al., 2018).

#### 3.6.2 Phage release and antibacterial activity of the hydrogel

**FIGURE 7:**
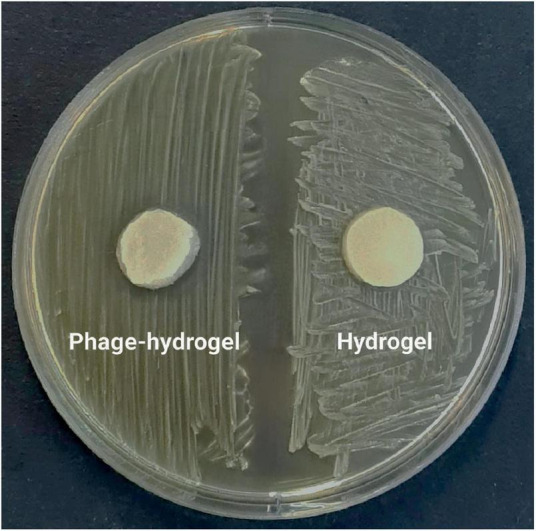
The inhibitory effect of the phage-loaded hydrogel on bacterial growth demonstrated by the inhibition zone in the disk diffusion assay.

Consistent with the results in Figure 6B, the phage-loaded hydrogel effectively inhibited bacterial growth in the broth culture medium. In contrast, the control group represented a significant increase in turbidity after 24 hours which suggests the effective release of the phage from the hydrogel. The disk diffusion assay further confirmed these results. A clear halo zone surrounding the phage-containing hydrogel disk indicated bacterial inhibition by the released phage particles. In contrast, the empty hydrogel disk, which served as a negative control, revealed no halo formation (Figure 7).

#### 3.6.3 SEM analysis

**FIGURE 8:**
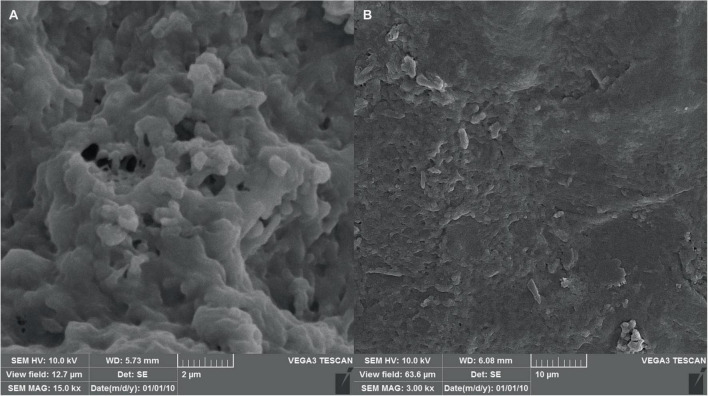
SEM micrographs of the hydrogel with scale bars of **(A)** 2 μm and **(B)** 10 μm, indicating successful formation of the hydrogel structure as well as proper shape and size.

The morphology of the hydrogel at different magnifications using scanning electron microscopy has been shown in Figure 8. The image reveals a continuous, interconnected porous structure, possibly formed by a combination of phase separation during hydrogel formation and sublimation of water removed by the freezing process. The observed pores have an average diameter of approximately 80 nm, suggesting a potential applicability for phage encapsulation.

#### 3.6.4 FTIR spectroscopy

**FIGURE 9:**
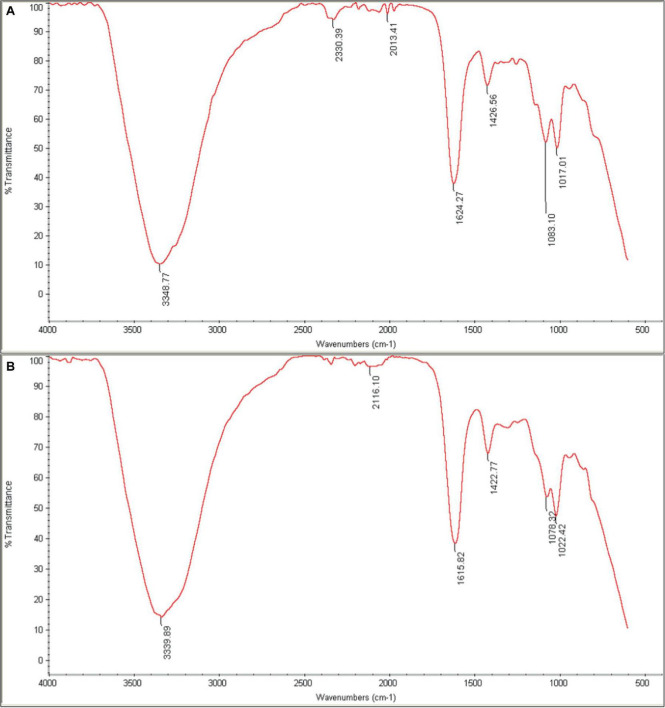
FTIR spectra of **(A)** phage-loaded hydrogel and **(B)** control hydrogel. The peaks in the range 3200–3500 cm^–1^ are related to (O–H), those in the 1620–1420 cm^–1^ range to (C=O and C–N) bonds, and at 1020 cm^–1^ to (C–O) bonds. The presence of the distinctive 2330 cm^–1^ peak in the phage-loaded hydrogel spectra indicated the possible retention of phage in the hydrogel.

FTIR was used to characterize the chemical composition of the hydrogel, both empty and loaded with phage as shown in Figure 9. The spectra were consistent with previous studies on similar hydrogels (Zhang et al., 2021). The broad peak in the range of 3200–3500 cm^–1^ corresponds to the stretching vibration of O-H bonds, which are likely due to inter- and intramolecular hydrogen bonding in the polysaccharides (sodium alginate, CMC and hyaluronic acid) within the hydrogel. The peaks at approximately 1620 cm^–1^ and 1420 cm^–1^ can be attributed to the amide I vibrations (C = O stretching) of carboxyl groups and the stretching vibrations of primary amine bonds (C-N), respectively. Finally, the peak observed at about 1020 cm^–1^ is assigned to the C-O stretching vibration of primary alcohols. The intensity of the peak at 2330 cm^–1^ appears to be higher in the phage-loaded hydrogel. While this wavenumber may indicate primary or secondary amines in protein structures, it could possibly provide evidence for phage encapsulation in the hydrogel.

### 3.7 *In vivo* results

#### 3.7.1 Wound healing process

**FIGURE 10:**
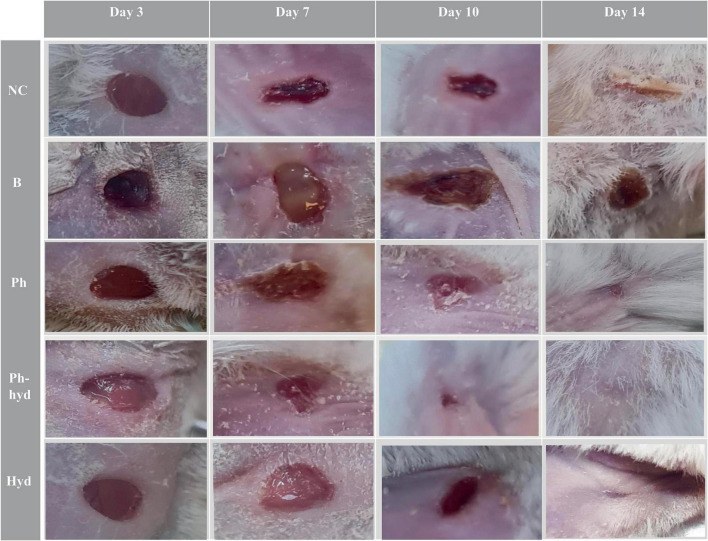
Visualizing wound healing efficacy across treatment groups. The wound healing progress is shown in five different treatment groups over a 14-day period (days 3, 7, 10, and 14 are illustrated). A progression is evident, with the phage hydrogel group (Ph-hyd) demonstrating a clear wound closure. Notably, the (Ph) group also exhibited an obvious healing effect, ranking second in wound closure efficacy. NC: Negative control. B: Positive control. Ph: Phage-treated group. Ph-hyd: Phage hydrogel group. Hyd: Hydrogel control group.

Wound closure and epithelialization were monitored in all six groups of mice (Figure 10). Results demonstrated significant wound healing in the phage-loaded hydrogel group (Ph-hyd) compared to all other groups. Complete wound closure was observed in the Ph-hyd group by day 14, with healing starting as early as day 3. In addition, the phage-treated group (Ph) showed a faster healing process compared to the wounded control group (NC) and the untreated infectious group (B), achieving significant wound closure by day 14. However, the healing rate was slower than in the Ph-hyd group, suggesting that although phage therapy promotes wound healing, its efficacy is optimized by a suitable delivery system such as the hydrogel. The NC group displayed delayed wound closure compared to the Ph and Ph-hyd groups, with a remaining large wound on day 10. The slowest healing rate was observed in the B group, highlighting the detrimental effects of untreated infection on wound healing.

#### 3.7.2 Histopathological studies

**FIGURE 11:**
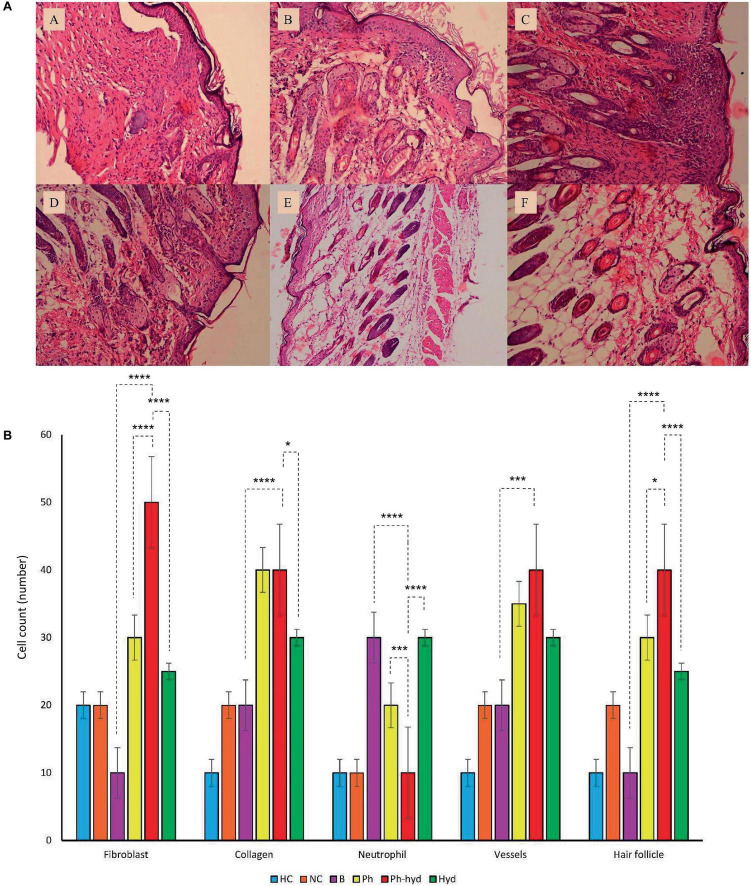
**(A)** Histopathological sections of skin obtained on 14^th^ day post wound infection (A: NC, B: HC, C: B, D: Ph, E: Ph-hyd, F: Hyd). **(B)** The neutrophil, fibroblast, blood vessel, and hair follicle counts and the percentage of collagen growth as markers for the evaluation of the wound healing process in all experimental groups. One-way ANOVA test showed that the differences among some groups are statistically significant (*P* value < 0.05). The p-values of fibroblast, collagen, neutrophil, vessels, hair follicle among all vs. all groups have been shown in Supplementary Table 7. The p-values of Ph-hyd vs. B, Ph-hyd vs. Ph and Ph-hyd vs. Hyd, group were as follows: < 0.0001, < 0.0001 and < 0.0001 (for fibroblast), < 0.0001, 0.9361 and 0.0111 (for collagen), < 0.0001, 0.0003 and < 0.0001 (for neutrophil) *p-value < 0.05, ***p-value < 0.001, and ****p-value < 0.0001, 0.0002, 0.2123 and 0.1271 (for vessel) and < 0.0001, 0.0179 and < 0.0001 (for hair follicle) respectively. The underlined P-values are not significant.

Histopathologic analysis of tissue samples collected from the wounds on day 14 revealed incomplete epidermal regeneration in the positive control (B) and negative control (NC) groups. These groups displayed significantly increased epidermal thickness compared to the phage-loaded hydrogel group (Ph-hyd). In contrast, the healthy negative control (HC) and the Ph-hyd groups exhibited normal epidermal thickness, indicating efficient epidermal repair (Figure 11A). Interestingly, hair follicles and blood vessels in the hydrogel-treated groups (Ph-hyd and hyd) displayed a morphology closely resembling those observed in healthy skin. They promote the restoration of essential skin structures, potentially contributing to the accelerated healing and epithelialization observed in these groups Fibroblast density, a marker of skin regeneration and proliferation, was significantly higher in the Ph-hyd and phage-treated (Ph) groups compared to the control groups. These results support the positive impact of phage therapy in promoting tissue regeneration. In addition, the presence of neutrophils, a marker of inflammation and potential infection, was significantly lower in the Ph-hyd group compared to the positive control group. This indicates that the hydrogel delivery system was effective in reducing inflammation within the wound area. At the same time, the results indicate the highest level of inflammation for the control group (Figure 11B).

## 4 Discussion

*E. faecium* is one of the main causes of hospital-acquired infections. Owing to its resistance to antibiotics treating this bacterium in hospital settings has become challenging (Peng et al., 2022). The rise in superbugs in clinical settings, accentuates the need for the development of innovative treatment approaches to effectively address the escalating threat of antimicrobial resistance. Among these novel approaches, phage therapy has emerged as an encouraging method for combating antibiotic-resistant bacteria.

To ensure the successful performance of the isolated phage as an antibacterial tool, we investigated its key properties through a series of experiments. EF-M80 exhibited strong lytic activity, a proper host spectrum, anti-biofilm activity and acceptable stability under environmental conditions. Also, analysis of the phage life cycle through the lens of adsorption rate, one-step growth curve, and MOI determination offers valuable perceptions into critical parameters like burst size, latent period, and optimal lysis time. Burst size reflects the average number of progeny phages released per infected host cell (Kannoly et al., 2023). Its variability across different phages highlights the unique replication efficiency. Latent period signifies the duration between phage adsorption to the host and the subsequent release of new phages through cell lysis. This parameter essentially represents the time required for the intracellular replication cycle of the phage to complete (Abedon et al., 2003). The burst size and latent period observed for the EF-M80 phage were similar to the previously reported *Enterococcus* phages (Abed et al., 2024).

Adsorption rate provides vital information regarding the efficiency and speed of the early stages of infection, particularly phage-host recognition and attachment (Grygorcewicz et al., 2022). The high adsorption rate of the EF-M80 phage indicates a strong binding affinity between its ligands and the corresponding receptors on the bacterial cell surface. This translates to a faster initiation of the infection cycle, potentially contributing to the effectiveness, survival, and competitive advantage of the phage over other phages or host defense mechanisms (Gallet et al., 2009).

Genome sequencing of the EF-M80 phage genome revealed that there are no genes encoding proteins associated with the lysogenic cycle like integrase, recombinase, repressors, or excisionase. These genes are key markers of temperate viruses (Necel et al., 2020). The finding supports the hypothesis that EF-M80 preferentially undergoes the lytic cycle, minimizing the risk of lysogenic transformation in phage therapy applications. This property may be advantageous in phage therapy where rapid elimination of bacteria is desired.

Before initiating phage therapy, it is essential to perform whole-genome sequencing of newly isolated bacteriophages to identify missing resistance and virulence genes to ensure safety. This approach helps optimize treatment outcomes while minimizing resource consumption (Liu et al., 2022). In this study, the potential of bacteriophage EF-M80 to act as a vector for horizontal gene transfer (HGT) of antibiotic resistance genes (ARGs) and virulence factors was investigated. While bacteriophages are known facilitators of HGT (Wójcicki et al., 2021), we did not identify any ARGs or virulence factor genes within the EF-M80 genome.

EF-M80 encodes a set of lytic proteins, including endolysins, XhlA-like hemolysin, and holin (ORFs 18, 19, 20, and 21), which render its potency as an antibacterial product. In the cytoplasmic membrane, holins form micron-sized pores that allow endolysins to penetrate and degrade the peptidoglycan. Eventually, the bacterial cytoplasmic membrane is ruptured by the endopeptidase (Zaki et al., 2023). Some bacteriophages, like those we studied, have a holin-like gene in the hemolytic cassette that encodes a protein with an XhlA domain, which is characteristic of the hemolytic process. In *Enterococcus* phages, XhlA is always associated with the holin, presumably forming a membrane complex required for endolysin release across the membrane (Viglasky et al., 2023).

Additionally, the comparative genomic analysis revealed that EF-M80 shares a similar genomic structure with previously characterized phages, but with variations in non-coding regions. Notably, pan/core analysis of 56 genomes revealed that proteins involved in lysis, membrane protein, tail tape measure protein, and portal protein were the core of bacteriophages and had single or multiple amino acid polymorphisms. Interestingly, a substantial portion of the phage proteome displayed diversity in presence/absence across different phages. These findings highlight the importance of protein-level comparisons for establishing phylogenetic relationships among bacteriophages. Unlike DNA-based methods, which can be confounded by genomic mosaicism arising from frequent recombination and segment exchange, protein profiles offer a meaningful comparison for phage phylogeny (Khot et al., 2020). Intriguingly, this study showed that the tertiary structure of endolysin of the EF-M80 phage is very similar to chain A of the LytA protein from *Streptococcus pneumonia* (data have not shown). It appears that the source of the endolysin family proteins can be traced back to bacteriophages and that their integration into the bacterial chromosome occurs during the transduction process.

The growing prevalence of multidrug-resistant (MDR) *Enterococcus* spp. infections in soft tissues, wounds, and surgical sites necessitates the exploration of novel therapeutic strategies (Rajkumari et al., 2014). Biomaterials have emerged as promising tools for controlled drug delivery, and in particular hydrogels, show great potential as wound dressings. This study investigates the efficacy of a novel delivery system−encapsulated bacteriophages in a sodium alginate-based hydrogel – for treating murine wound infections caused by *E. faecium*. Hydrogels offer a near-ideal environment for wound healing. They maintain a moist environment, facilitate gas exchange, and exhibit biocompatibility, promoting optimal healing conditions (Jacob et al., 2021). The hydrogel composition and crosslinking chemistry played a significant role in preserving phage infectivity during storage. The hydrated nano-fibrous network, optimized porosity and mild crosslinking conditions provided a favorable microenvironment within the hydrogels to entrap and stabilize phage particles while retaining their antimicrobial function. Additionally, the amphoteric character of the phage allowed for easy mixing with negatively charged polysaccharides like alginate, facilitating the encapsulation process (Rana and De la Hoz Siegler, 2024). Previous reports have shown that hydrogels with similar formulations to ours can maintain phage stability for at least 2 weeks (Shafigh Kheljan et al., 2023). These findings support the idea that the hydrogel microenvironment can influence phage stability.

The current hydrogel formulation leverages a three-component design, each component contributing specific functionalities. Sodium alginate serves as a carrier for therapeutic agents, ensuring their targeted delivery to the wound site. Sodium alginate plays a crucial role in optimizing the properties of hydrogel (Lee and Mooney, 2012). Carboxymethyl cellulose (CMC) enhances the hydrogel’s functionality by imparting superabsorbent properties, thermal stability, and the formation of interconnected pores within the structure, facilitating efficient phage encapsulation (Tuan Mohamood et al., 2021). Hyaluronic acid (HA), the third element of the hydrogel, is a core component of the extracellular matrix and significantly impacts wound healing processes. During inflammation, HA fragments bind to fibrinogen, initiating clotting and creating a scaffold for immune cell migration (Frenkel, 2014). Studies have shown that exogenous HA enhances re-epithelialization, highlighting its importance in tissue repair (Nyman et al., 2019). The observed improvements in granulation, collagen deposition, fibroblast presence, and epithelialization in the hydrogel-treated groups can be attributed to the multifaceted roles of HA in wound healing.

Beyond alginate-based hydrogels, various formulations and production methods have been explored for developing phage-delivering hydrogels. These include materials such as polyethylene glycol (PEG), polyvinyl alcohol (PVA), hydroxypropyl methylcellulose (HPMC), and agarose-hyaluronic acid methacrylate (HAMA). These alternative hydrogels hold promise for treating a range of infections, including implant-related osteomyelitis, urinary tract infections from catheters, and trauma-associated wound infections (Kim et al., 2021). Studies have demonstrated the use of PVA hydrogels for catheter blockage prevention. Milo et al. designed a surface coating PVA hydrogel loaded with phage to target biofilms formed by *Proteus mirabilis* (Milo et al., 2017). Alginate-nanohydroxyapatite hydrogels loaded with bacteriophage have also shown efficacy. Barros et al. reported that these hydrogels reduced the ability of multidrug-resistant *E. faecalis* to adhere and form colonies in femoral tissues following bone graft implantation (Barros et al., 2020). Combination therapy using phages and antibiotics (phage-antibiotic synergy, PAS) has also been explored. Mukhopadhyay et al. employed an HPMC hydrogel containing a specific phage (vB_AbaM-IME-AB2) and the antibiotic colistin to eradicate both biofilm and planktonic forms of *Acinetobacter baumannii* (Mukhopadhyay et al., 2023).

The *in vivo* histopathological evaluation demonstrated that EF-M80 phage retained its antibacterial properties, effectively lysing *E. faecium* within the host environment. On the other hand, encapsulation of the phage within a hydrogel delivery system significantly enhanced its efficacy in promoting wound healing in wound-infected mice models. These findings collectively suggest the promising potential of this phage-based therapeutic method for the treatment of infections associated with antibiotic-resistant *E. faecium*.

## 5 Conclusion

Increasing antibiotic resistance of the ESKAPE bacteria causing hospital-acquired infections is alarmingly raised as a health problem all over the world. Until now, to prevent the antimicrobial resistance, the use of various methods, and strategies has been suggested. Bacteriophages seem to be efficient and attractive tools against antibiotic-resistant bacteria. In this study, EF-M80 phage revealed that it can be used against *E. faecium* in terms of promising physicochemical and genetic characteristics. The stability of the EF-M80 phage to environmental conditions and its lysis property on *E. faecium* highlighted that it could be a promising tool against this bacterium. Furthermore, the genome sequencing of *Enterococcus* phage strain EF-M80 revealed lysis cassettes that act cooperatively to mediate phage-induced host cell lysis. The phage encapsulated in the hydrogel texture represented a synergistic effect on the wound healing process. This study may provide the basis for the future use of hydrogel-encapsulated EF-M80 phage in curing biofilm related *E. faecium* infections.

## Data availability statement

The Enterococcus phage strain EF-M80 was deposited in the GenBank database under accession number OR767211. In addition, all bacteriophage genomes considered in this study were retrieved from the GenBank database. The accession number(s) can be found in the article/Supplementary material.

## Ethics statement

The requirement of ethical approval was waived by this project was done based on ethical guidelines as previously approved by the Al-Zahra University (Ir.ALZAHRA.REC.1401.015). for the studies on humans because samples without names and codes. The studies were conducted in accordance with the local legislation and institutional requirements. Written informed consent for participation was not required from the participants or the participants’ legal guardians/next of kin in accordance with the national legislation and institutional requirements. The human samples used in this study were acquired from primarily isolated as part of your previous study for which ethical approval was obtained. The animal study was approved by this project was done based on ethical guidelines as previously approved by the Al-Zahra University (Ir.ALZAHRA.REC.1401.015).

## Author contributions

MK: Data curation, Formal analysis, Software, Visualization, Writing−original draft. AE: Conceptualization, Project administration, Supervision, Validation, Writing−review and editing. SA: Data curation, Formal analysis, Writing−review and editing. MS: Project administration, Supervision, Validation, Writing−review and editing. SB: Data curation, Writing−review and editing. HS: Data curation, Formal analysis, Writing−review and editing. FB: Data curation, Formal analysis, Software, Writing−review and editing. AS: Data curation, Writing−review and editing.
