# Supplementary material for: A novel Enterococcus faecium phage EF-M80: unveiling the effects of hydrogel-encapsulated phage on wound infection healing
Source: Front Microbiol. 2024 Jun 28;15:1416971. doi: 10.3389/fmicb.2024.1416971 (PMC11239553; doi:10.3389/fmicb.2024.1416971)
Supplement: Supplementary file 1 [file Data_Sheet_1.docx]

**Supplementary Data:**

**Table S1**. Percentage of resistance of bacterial isolates to various antibiotics.

| **Antibiotic** | **Resistant Enterococcus faecium**  **(N=50) (%)** |
| --- | --- |
| Ampicillin-sulbactam | 46 (92%) |
| Gentamicin | 46 (92%) |
| Streptomycin | 50 (100%) |
| Vancomycin | 50 (100%) |
| Teicoplanin | 35 (70%) |
| Chloramphenicol | 8 (16%) |
| Linezolid | 3 (6%) |
| Rifampin | 50 (100%) |
| Erythromycin | 50 (100%) |
| Tetracycline | 36 (72%) |
| Ciprofloxacin | 50 (100%) |

**Table S2**. Tukey's multiple comparisons test using ANOVA analysis among different groups of environmental tests. The groups highlighted in red are statistically non-significant.

**Temperature**

| **Tukey's multiple comparisons test** | **Mean Diff.** | **95% CI of diff.** | **Significant?** | **Summary** | **P Value** |
| --- | --- | --- | --- | --- | --- |
| **-20 vs. 4** | -1.433 | -1.984 to -0.8829 | Yes | **** | < 0.0001 |
| **-20 vs. 20** | -5.400 | -5.950 to -4.850 | Yes | **** | < 0.0001 |
| **-20 vs. 37** | -5.667 | -6.217 to -5.116 | Yes | **** | < 0.0001 |
| **-20 vs. 50** | -2.333 | -2.884 to -1.783 | Yes | **** | < 0.0001 |
| **-20 vs. 60** | 1.333 | 0.7829 to 1.884 | Yes | **** | < 0.0001 |
| **-20 vs. 70** | 1.333 | 0.7829 to 1.884 | Yes | **** | < 0.0001 |
| **-20 vs. 80** | 1.333 | 0.7829 to 1.884 | Yes | **** | < 0.0001 |
| **4 vs. 20** | -3.967 | -4.517 to -3.416 | Yes | **** | < 0.0001 |
| **4 vs. 37** | -4.233 | -4.784 to -3.683 | Yes | **** | < 0.0001 |
| **4 vs. 50** | -0.9000 | -1.450 to -0.3496 | Yes | *** | 0.0007 |
| **4 vs. 60** | 2.767 | 2.216 to 3.317 | Yes | **** | < 0.0001 |
| **4 vs. 70** | 2.767 | 2.216 to 3.317 | Yes | **** | < 0.0001 |
| **4 vs. 80** | 2.767 | 2.216 to 3.317 | Yes | **** | < 0.0001 |
| **20 vs. 37** | -0.2667 | -0.8171 to 0.2838 | No | ns | 0.7006 |
| **20 vs. 50** | 3.067 | 2.516 to 3.617 | Yes | **** | < 0.0001 |
| **20 vs. 60** | 6.733 | 6.183 to 7.284 | Yes | **** | < 0.0001 |
| **20 vs. 70** | 6.733 | 6.183 to 7.284 | Yes | **** | < 0.0001 |
| **20 vs. 80** | 6.733 | 6.183 to 7.284 | Yes | **** | < 0.0001 |
| **37 vs. 50** | 3.333 | 2.783 to 3.884 | Yes | **** | < 0.0001 |
| **37 vs. 60** | 7.000 | 6.450 to 7.550 | Yes | **** | < 0.0001 |
| **37 vs. 70** | 7.000 | 6.450 to 7.550 | Yes | **** | < 0.0001 |
| **37 vs. 80** | 7.000 | 6.450 to 7.550 | Yes | **** | < 0.0001 |
| **50 vs. 60** | 3.667 | 3.116 to 4.217 | Yes | **** | < 0.0001 |
| **50 vs. 70** | 3.667 | 3.116 to 4.217 | Yes | **** | < 0.0001 |
| **50 vs. 80** | 3.667 | 3.116 to 4.217 | Yes | **** | < 0.0001 |
| **60 vs. 70** | 0.0 | -0.5504 to 0.5504 | No | ns | > 0.9999 |
| **60 vs. 80** | 0.0 | -0.5504 to 0.5504 | No | ns | > 0.9999 |
| **70 vs. 80** | 0.0 | -0.5504 to 0.5504 | No | ns | > 0.9999 |

**Concentration of NaCl (%)**

| **Tukey's multiple comparisons test** | **Mean Diff.** | **95% CI of diff.** | **Significant?** | **Summary** | **P Value** |
| --- | --- | --- | --- | --- | --- |
| 5% vs. 10% | 0.9000 | -0.3130 to 2.113 | No | ns | 0.1358 |
| 5% vs. 15% | 2.167 | 0.9536 to 3.380 | Yes | ** | 0.0037 |
| 10% vs. 15% | 1.267 | 0.05364 to 2.480 | Yes | * | 0.0423 |

**pH**

| **Tukey's multiple comparisons test** | **Mean Diff.** | **95% CI of diff.** | **Significant?** | **Summary** | **P Value** |
| --- | --- | --- | --- | --- | --- |
| **2 vs. 4** | -10.53 | -11.30 to -9.767 | Yes | **** | < 0.0001 |
| **2 vs. 7** | -7.000 | -7.766 to -6.234 | Yes | **** | < 0.0001 |
| **2 vs. 10** | -9.267 | -10.03 to -8.500 | Yes | **** | < 0.0001 |
| **2 vs. 14** | 0.0 | -0.7664 to 0.7664 | No | ns | > 0.9999 |
| **4 vs. 7** | 3.533 | 2.767 to 4.300 | Yes | **** | < 0.0001 |
| **4 vs. 10** | 1.267 | 0.5003 to 2.033 | Yes | ** | 0.0020 |
| **4 vs. 14** | 10.53 | 9.767 to 11.30 | Yes | **** | < 0.0001 |
| **7 vs. 10** | -2.267 | -3.033 to -1.500 | Yes | **** | < 0.0001 |
| **7 vs. 14** | 7.000 | 6.234 to 7.766 | Yes | **** | < 0.0001 |
| **10 vs. 14** | 9.267 | 8.500 to 10.03 | Yes | **** | < 0.0001 |

**Table S3**. Phage dilutions for biofilm eradication.

| **Biofilm** | **Phage dilution** |
| --- | --- |
| 1-day biofilm | 10^5^-fold |
| 3-day biofilm | 10^4^-fold |
| 5-day biofilm | 10^3^-fold |

**Table S4**. Data on 56 genomes of *Enterococcus* phages isolated with different characteristics. The coverage and identity of six core genes among genomes have shown in numbers based on percent.

| Accession number | Name | Date | Organism | DNA | Sequencing Technology | Country | Host | Isolation source | Portal protein | Tail tape measure protein | XhlA-like hemolysin | Holin | Endolysin | Membrane protein |
| --- | --- | --- | --- | --- | --- | --- | --- | --- | --- | --- | --- | --- | --- | --- |
| OP254195 | vB_EfS_L1 | 22-Oct-22 | Efquatrovirus | Linear | Illumina | China | *E. faecalis* | ND | 100, 99 | 100, 96 | 100, 98 | 100, 98 | 100, 98 | 100, 97 |
| OP172797 | EF326P1 | 27-Dec-22 | Efquatrovirus | Linear | Illumina | China | *E. faecalis* | Municipal sewage | 100, 99 | 100, 98 | 100, 100 | 100, 97 | 49, 90 | 92, 100 |
| MZ272341 | vB_EfaS_785CC | 22-Sep-21 | Efquatrovirus | Linear | IonTorrent | China | *E. faecalis* | ND | 100, 100 | 100, 98 | 100, 100 | 100, 98 | 49, 90 | 92, 100 |
| MZ182246 | vB_EfaS_785CS | 29-May-21 | Efquatrovirus | Linear | IonTorrent | China | *E. faecalis* | ND | 100, 100 | 54, 97 | 100, 100 | 100, 98 | 49, 90 | 92, 100 |
| ON113334 | ZXL | 24-Mar-23 | Efquatrovirus | Circular | ND | China | *E. faecalis* | Sewage sample | 100, 99 | 100, 98 | 100, 98 | 100, 96 | 49, 90 | 92, 98 |
| OQ269649 | DEfc27b | 15-Feb-23 | Efquatrovirus | Linear | ND | China | *E. faecalis* | Water | 100, 99 | 100, 98 | 100, 100 | 100, 97 | 49, 90 | 92, 98 |
| MT829326 | FX417 | 1-Feb-21 | Efquatrovirus | Linear | Illumina | China | *E. faecalis* | Mouse gut | 100, 99 | 77, 97 | 100, 100 | 100, 97 | 49, 90 | 92, 98 |
| MT520979 | vB_EfaS-271 | 1-Sep-20 | Efquatrovirus | Linear | Illumina | Poland | *E. faecalis* | Sewage | 100, 100 | 100, 99 | 100, 97 | 100, 98 | 100, 98 | 100, 92 |
| MH355583 | vB_EfaS_LM99 | 21-Jul-18 | Efquatrovirus | Linear | ND | Portugal | *E. faecalis* | Raw sewage, wastewater treatment plant | 100, 99 | 100, 98 | 100, 100 | 100, 98 | 100, 98 | 92, 98 |
| OR237564 | vB_Efa_VP15 | 20-Sep-23 | Efquatrovirus | Linear | Illumina | Slovakia | *E. faecalis* | Wastewater | 100, 98 | 100, 98 | 81, 89 | 100, 98 | 100, 97 | 92, 100 |
| MG264739 | phiNASRA1 | 6-May-20 | Efquatrovirus | Linear | Illumina | USA | *E. faecalis* | Greenwood WWTP | 99, 99 | 100, 91 | 100, 97 | 98, 75 | 44, 86 | 100, 85 |
| ON113176 | vB_OCPT_CCS4 | 22-May-22 | Efquatrovirus | Linear | Illumina | USA | Enterococcus sp. | Sewage | 100, 98 | 100, 96 | 100, 95 | 98, 76 | 100, 97 | 100, 79 |
| OR237563 | vB_Efa_VP14 | 20-Sep-23 | Efquatrovirus | Linear | Illumina | Slovakia | *E. faecalis* | Wastewater | 100, 98 | 100, 96 | 100, 95 | 98, 76 | 100, 97 | 100, 88 |
| OP889240 | vB_EfaS_Ef6.1 | 27-Dec-22 | Efquatrovirus | Linear | Illumina | China | *E. faecalis* | Sewage treatment plants | 100, 90 | 100, 62 | 100, 96 | 98, 76 | 44, 85 | 100, 91 |
| MK721190 | vB_EfaS_Ef6.4 | 17-Apr-19 | Efquatrovirus | Circular | ND | USA | *E. faecalis* | Raw sewage water obtained from a North City Water Reclamation Plant | 97, 69 | 53, 83 | 81, 89 | 100, 98 | 100, 96 | ND |
| MH375074 | LY0323 | 7-Jul-18 | Efquatrovirus | Linear | Illumina | China | *E. faecalis* | ND | 100, 98 | 100, 61 | 100, 96 | 98, 76 | 100, 99 | 100, 83 |
| MZ326865 | Sigurd | 3-Feb-22 | Efquatrovirus | Linear | Illumina | USA | *E. faecalis* | Wastewater collected | 100, 90 | 100, 62 | 100, 97 | 98, 72 | 44, 85 | 100, 91 |
| OP559177 | vB_Efa29212_2e | 12-Oct-22 | Efquatrovirus | Linear | Illumina | Poland | Enterococcus sp. | ND | 100, 98 | 99, 95 | 100, 95 | 98, 76 | 100, 94 | 100, 79 |
| MK721191 | vB_EfaS_Ef5.4 | 17-Apr-19 | Efquatrovirus | Linear | ND | USA | *E. faecalis* | Raw sewage water obtained from a North City Water Reclamation Plant | 100, 99 | 100, 95 | 100, 93 | 98, 76 | 100, 96 | 100, 89 |
| MK721187 | vB_EfaS_Ef6.1 | 17-Apr-19 | Efquatrovirus | Circular | ND | USA | *E. faecalis* | Raw sewage water obtained from a North City Water Reclamation Plant | 100, 90 | 100, 62 | 100, 97 | 98, 76 | 44, 85 | 100, 91 |
| KF728385 | IME_EF3 | 27-Aug-14 | Efquatrovirus | Linear | IonTorrent | China | *E. faecalis* | Sewage | 100, 90 | 100, 62 | 100, 97 | 98, 73 | 44, 84 | 100, 92 |
| MK721199 | vB_EfaS_Ef5.1 | 17-Apr-19 | Efquatrovirus | Linear | ND | USA | *E. faecalis* | Raw sewage water obtained from a North City Water Reclamation Plant | 100, 99 | 100, 96 | 81, 89 | 98, 76 | 100, 95 | 100, 89 |
| MK721200 | vB_EfaS_Ef5.3 | 17-Apr-19 | Efquatrovirus | Linear | ND | USA | *E. faecalis* | Raw sewage water obtained from a North City Water Reclamation Plant | 100, 98 | 100, 95 | 81, 89 | 100, 98 | 100, 96 | 100, 89 |
| MT661598 | vB_EFaS_TV54 | 1-Feb-21 | Efquatrovirus | Linear | Illumina | Italy | *E. faecalis* | Municipal wastewater | 100, 98 | 100, 96 | 100, 96 | 98, 76 | 47, 92 | 100, 89 |
| OL539449 | vB_EfaS_Paulomi | 15-Dec-21 | Efquatrovirus | Linear | ND | USA | *E. faecalis* | Raw sewage | 100, 99 | 99, 96 | 81, 86 | 100, 96 | 100, 95 | 100, 79 |
| OR237565 | vB_Efa_VP16 | 20-Sep-23 | Efquatrovirus | Linear | Illumina | Slovakia | *E. faecalis* | Wastewater | 100, 99 | 100, 96 | 100, 95 | 100, 77 | 44, 85 | 100, 88 |
| MT661597 | vB_EFaS_TV51 | 1-Feb-21 | Efquatrovirus | Linear | Illumina | Italy | *E. faecalis* | Municipal wastewater | 100, 99 | 100, 96 | 100, 95 | 100, 77 | 47, 89 | 100, 91 |
| JX193904 | EfaCPT1 | 28-Jul-12 | Efquatrovirus | Circular | Sanger dideoxy sequencing; 454 | USA | *E. faecalis* | Sewage, college station, tx, usa | 100, 99 | 100, 96 | 100, 96 | 98, 76 | 44, 85 | 100, 89 |
| KF733017 | IME-EF4 | 14-Dec-13 | Efquatrovirus | Linear | IonTorrent | China | *E. faecalis* | Sewage | 100, 98 | 100, 60 | 100, 93 | 100, 94 | 100, 98 | 100, 83 |
| OL799257 | phiSHEF11 | 31-Mar-22 | Efquatrovirus | Linear | Illumina | UK | *E. faecalis* | Wastewater | 100, 99 | 100, 95 | 100, 97 | 100, 94 | 100, 96 | 100, 80 |
| MT857001 | EFA1 | 12-Oct-20 | Efquatrovirus | Linear | ND | Australia | *E. faecalis* | ND | 100, 97 | 100, 94 | 100, 97 | 98, 75 | 44, 84 | 100, 85 |
| MT627482 | vB_EFaS_TV217 | 1-Feb-21 | Efquatrovirus | Linear | Illumina | Italy | *E. faecalis* | Municipal wastewater | 100, 98 | 100, 96 | 100, 96 | 98, 76 | 44, 85 | 100, 85 |
| OL799256 | phiSHEF10 | 31-Mar-22 | Efquatrovirus | Linear | Illumina | UK | *E. faecalis* | Wastewater | 100, 99 | 100, 96 | 100, 95 | 98, 75 | 44, 85 | 100, 88 |
| MK721186 | vB_EfaS_Ef5.2 | 17-Apr-19 | Efquatrovirus | Linear | ND | USA | *E. faecalis* | Raw sewage water obtained from a North City Water Reclamation Plant | 100, 99 | 100, 96 | 100, 96 | 98, 76 | 47, 92 | 100, 88 |
| OR052631 | Ef212 | 30-Jul-23 | Efquatrovirus | Linear | ND | Turkey | *E. faecalis* | Sewage water | 100, 99 | 100, 94 | 81, 90 | 100, 98 | 47, 92 | 100, 80 |
| MK360024 | vB_EfaS_Max | 6-Feb-19 | Efquatrovirus | Linear | ND | Portugal | *E. faecalis* | Raw sewage from wastewater treatment plant | 100, 99 | 100, 96 | 100, 96 | 98, 76 | 44, 84 | 100, 88 |
| OR360270 | SSMH01 | 4-Oct-23 | Efquatrovirus | Linear | Illumina | Pakistan | *E. faecalis* | ND | 100, 99 | 100, 96 | 80, 89 | 98, 75 | 44, 85 | 100, 79 |
| KT932701 | vB_EfaS_IME196 | 7-Jul-16 | Efquatrovirus | Circular | IonTorrent | China | *E. faecalis* | Hospital sewage | 100, 98 | 100, 84 | 81, 89 | 98, 73 | 44, 85 | ND |
| KR131750 | Ec-ZZ2 | 2-Sep-16 | Efquatrovirus | Linear | Illumina | China | faecium | Hospital sewage | 100, 98 | 100, 60 | 80, 89 | 100, 96 | 100, 96 | 100, 83 |
| OQ866599 | SSMH02 | 30-Jul-23 | Efquatrovirus | Linear | Illumina | Pakistan | *E. faecalis* | Wastewater | 100, 99 | 100, 96 | 100, 96 | 98, 73 | 44, 84 | 100, 85 |
| LC623721 | vB_EfaS-SRH2 | 6-Apr-21 | Efquatrovirus | Linear | ND | Iran | *E. faecalis* | ND | 100, 99 | 100, 91 | 81, 89 | 100, 98 | 47, 93 | 100, 88 |
| MK982307 | MSF2 | 2-May-20 | Efquatrovirus | Linear | Sanger dideoxy sequencing; 454 | Slovakia | *E. faecalis* | ND | 97, 69 | 70, 59 | 81, 89 | 100, 98 | 100, 97 | ND |
| KX284704 | SANTOR1 | 2-Sep-16 | Efquatrovirus | Linear | IonTorrent | ND | *E. faecalis* | Raw domestic sewage | 100, 98 | 100, 96 | 100, 96 | 100, 94 | 100, 96 | 100, 80 |
| OL505084 | EFap02 | 11-Jan-22 | Efquatrovirus | Linear | Illumina | China | *E. faecalis* | Wastewater | 100, 90 | 100, 60 | 81, 87 | 100, 98 | 100, 96 | 100, 82 |
| MZ333461 | VEsP-2 | 8-Oct-21 | Efquatrovirus | Linear | Illumina | Viet Nam | Enterococcus sp. | River water | 100, 65 | 80, 59 | 81, 92 | 100, 92 | 100, 93 | ND |
| MT119359 | heks | 1-Feb-21 | Efquatrovirus | Linear | Illumina | Denmark | *E. faecalis* | Wastewater | 100, 65 | 80, 58 | 81, 92 | 100, 93 | 100, 93 | ND |
| MK125140 | Nonaheksakonaa | 1-Feb-21 | Efquatrovirus | Linear | Illumina | Denmark | *E. faecalis* | Wastewater | 100, 65 | 80, 58 | 81, 92 | 100, 93 | 100, 94 | ND |
| MF678788 | phiSHEF2 | 16-Sep-17 | Efquatrovirus | Linear | ND | ND | *E. faecalis* | Wastewater | 100, 99 | 100, 96 | 100, 95 | 98, 75 | 44, 85 | 100, 88 |
| MF678789 | phiSHEF4 | 16-Sep-17 | Efquatrovirus | Linear | Illumina | ND | *E. faecalis* | Wastewater | 100, 90 | 100, 95 | 100, 97 | 100, 95 | 100, 96 | 100, 85 |
| MF678790 | phiSHEF5 | 16-Sep-17 | Efquatrovirus | Linear | Illumina | ND | *E. faecalis* | Wastewater | 100, 99 | 100, 97 | 100, 95 | 98, 76 | 44, 85 | 100, 80 |
| MG708276 | PMBT2 | 2-Feb-18 | Efquatrovirus | Linear | Illumina | Germany | *E. faecalis* | Sewage | 100, 99 | 100, 96 | 100,95 | 98, 76 | 47, 89 | 100, 88 |
| MH193369 | LY0322 | 2-May-18 | Efquatrovirus | Linear | Illumina | China | *E. faecalis* | ND | 100, 99 | 100, 98 | 100, 98 | 100, 96 | 49, 90 | 92, 98 |
| MH203383 | vB_EfaS_AL3 | 6-Jun-19 | Efquatrovirus | Linear | ND | China | *E. faecalis* | Wastewater from the Second Hospital of Dalian Medical University | 100, 98 | 100, 98 | 100, 96 | 98, 76 | 100, 94 | 100, 94 |
| KJ127304 | AUEF3 | 17-Apr-14 | Efquatrovirus | Linear | Illumina | USA | *E. faecalis* | Processed sewage | 100, 99 | 54, 87 | 80, 90 | 100, 98 | 47, 93 | 100, 89 |
| MH203384 | vB_EfaS_AL2 | 8-May-18 | Efquatrovirus | Linear | Illumina | China | *E. faecalis* | Wastewater from the Second Hospital of Dalian Medical University | 100, 99 | 66, 90 | 81, 90 | 100, 93 | 100, 97 | 100, 83 |
| OR767211 | EF-M80 | 25-Nov-23 | Efquatrovirus | Linear | Illumina | Iran | faecium | Hospital sewage | 100, 100 | 100, 100 | 100, 100 | 100, 100 | 100, 100 | 100, 100 |

*ND means not determined

**Table S5**. Tukey's multiple comparisons test using ANOVA analysis among different group of swelling index group (Fig. 6A). The groups highlighted in red are statistically non-significant.

| **Tukey's multiple comparisons test** | **Mean Diff.** | **95% CI of diff.** | **Significant?** | **Summary** | **Adjusted P Value** |
| --- | --- | --- | --- | --- | --- |
| **1 vs. 2** | 2.300 | 0.2031 to 4.397 | Yes | * | 0.0305 |
| **1 vs. 3** | 0.7400 | -1.357 to 2.837 | No | ns | 0.7719 |
| **1 vs. 4** | 0.7400 | -1.357 to 2.837 | No | ns | 0.7719 |
| **1 vs. 24** | -6.290 | -8.387 to -4.193 | Yes | **** | < 0.0001 |
| **2 vs. 3** | -1.560 | -3.657 to 0.5369 | No | ns | 0.1794 |
| **2 vs. 4** | -1.560 | -3.657 to 0.5369 | No | ns | 0.1794 |
| **2 vs. 24** | -8.590 | -10.69 to -6.493 | Yes | **** | < 0.0001 |
| **3 vs. 4** | 0.0 | -2.097 to 2.097 | No | ns | > 0.9999 |
| **3 vs. 24** | -7.030 | -9.127 to -4.933 | Yes | **** | < 0.0001 |
| **4 vs. 24** | -7.030 | -9.127 to -4.933 | Yes | **** | < 0.0001 |

**Table S6**. Multiple T-test results for suppression of bacterial growth in liquid culture by the phage-loaded hydrogel (Fig. 6B).

|  | **Discovery?** | **P value** | **Significant?** | **Mean1** | **Mean2** | **Difference** | **SE of difference** | **t ratio** | **df** |
| --- | --- | --- | --- | --- | --- | --- | --- | --- | --- |
| **Beginning** |  | > 0.9999 | No | 0.09 | 0.09 | 0.0 | 0.00182574 | 0.0 | 4.0 |
| **Overnight** | * | < 0.0001 | Yes | 1.6 | 0.44 | 1.16 | 0.0464363 | 24.9804 | 4.0 |

**Table S7**. Tukey's multiple comparisons test using ANOVA analysis among different wound healing groups. The differences in groups that highlighted with green are statistically significant for all parameters and red highlights are statistically non-significant.

| **Tukey's multiple comparisons test** | **Fibroblast** | **Collagen** | **Neutrophil** | **Vessel** | **Hair follicle** |
| --- | --- | --- | --- | --- | --- |
| **HC vs. NC** | > 0.9999 | 0.0086 | 0.8366 | 0.0018 | 0.0005 |
| **HC vs. B** | 0.0102 | 0.0004 | < 0.0001 | 0.0094 | 0.9859 |
| **HC vs. Ph** | 0.0023 | < 0.0001 | 0.0002 | < 0.0001 | < 0.0001 |
| **HC vs. Ph-hyd** | < 0.0001 | < 0.0001 | 0.9992 | < 0.0001 | < 0.0001 |
| **HC vs. Hyd** | 0.3253 | < 0.0001 | < 0.0001 | < 0.0001 | 0.0008 |
| **NC vs. B** | 0.0102 | 0.4080 | < 0.0001 | 0.9079 | 0.0002 |
| **NC vs. Ph** | 0.0023 | < 0.0001 | 0.0010 | 0.0614 | 0.0048 |
| **NC vs. Ph-hyd** | < 0.0001 | < 0.0001 | 0.9526 | 0.0011 | < 0.0001 |
| **NC vs. Hyd** | 0.3253 | 0.0003 | < 0.0001 | 0.1063 | 0.9994 |
| **B vs. Ph** | < 0.0001 | < 0.0001 | 0.0845 | 0.0113 | < 0.0001 |
| **B vs. Ph-hyd** | < 0.0001 | < 0.0001 | < 0.0001 | 0.0002 | < 0.0001 |
| **B vs. Hyd** | 0.0003 | 0.0052 | 0.2414 | 0.0199 | 0.0003 |
| **Ph vs. Ph-hyd** | < 0.0001 | 0.9361 | 0.0003 | 0.2123 | 0.0179 |
| **Ph vs. Hyd** | 0.0802 | 0.0514 | 0.0017 | 0.9993 | 0.0028 |
| **Ph-hyd vs. Hyd** | < 0.0001 | 0.0111 | < 0.0001 | 0.1271 | < 0.0001 |
